# The impact of televised tobacco control advertising content on campaign recall: Evidence from the International Tobacco Control (ITC) United Kingdom Survey

**DOI:** 10.1186/1471-2458-14-432

**Published:** 2014-05-07

**Authors:** Sol Richardson, Ann McNeill, Tessa E Langley, Michelle Sims, Anna Gilmore, Lisa Szatkowski, Robert Heath, Geoffrey T Fong, Sarah Lewis

**Affiliations:** 1 UK Centre for Tobacco and Alcohol Studies, Division of Epidemiology and Public Health, University of Nottingham, Clinical Sciences Building, Nottingham City Hospital, Nottingham NG5 1 PB, UK; 2 UK Centre for Tobacco and Alcohol Studies, Institute of Psychiatry, King’s College London, 16 de Crespigny Park, London SE5 8AF, UK; 3 UK Centre for Tobacco and Alcohol Studies, Department for Health, University of Bath, Claverton Down, Bath BA2 7AY, UK; 4 School of Management, University of Bath, Claverton Down, Bath BA2 7AY, UK; 5 Department of Psychology, University of Waterloo, Waterloo, ON, Canada; 6 Ontario Institute for Cancer Research, Toronto, ON, Canada

**Keywords:** Tobacco control, Mass media campaigns, Recall, Emotive content

## Abstract

**Background:**

Although there is some evidence to support an association between exposure to televised tobacco control campaigns and recall among youth, little research has been conducted among adults. In addition, no previous work has directly compared the impact of different types of emotive campaign content. The present study examined the impact of increased exposure to tobacco control advertising with different types of emotive content on rates and durations of self-reported recall.

**Methods:**

Data on recall of televised campaigns from 1,968 adult smokers residing in England through four waves of the International Tobacco Control (ITC) United Kingdom Survey from 2005 to 2009 were merged with estimates of per capita exposure to government-run televised tobacco control advertising (measured in GRPs, or Gross Rating Points), which were categorised as either “positive” or “negative” according to their emotional content.

**Results:**

Increased overall campaign exposure was found to significantly increase probability of recall. For every additional 1,000 GRPs of per capita exposure to negative emotive campaigns in the six months prior to survey, there was a 41% increase in likelihood of recall (OR = 1.41, 95% CI: 1.24–1.61), while positive campaigns had no significant effect. Increased exposure to negative campaigns in both the 1–3 months and 4–6 month periods before survey was positively associated with recall.

**Conclusions:**

Increased per capita exposure to negative emotive campaigns had a greater effect on campaign recall than positive campaigns, and was positively associated with increased recall even when the exposure had occurred more than three months previously.

## Background

Tobacco control mass media campaigns have been shown to play a key role in encouraging smoking cessation among adults [1-5] and in reducing smoking prevalence [6]. In addition, there is growing evidence to suggest that campaigns featuring emotive or graphic content are more effective than those which do not [7-9]. While several studies have investigated youth recall of tobacco control advertising [10-13], only two to date have examined the impact of campaign content on recall among adults [14,15]. Both concluded that campaigns featuring graphic imagery or negative emotive content were more frequently recalled than those which did not. However, the first was based in Australia where the overwhelming majority of televised campaigns contain negative emotive content and graphic images [16,17] and the other was based on an internet survey rather than a representative sample of smokers.

Classic marketing theory assumes that high levels of recall improve advertising effectiveness and that campaign recall provides a proxy measure of effective campaign exposure [18,19]. Recent research calls this assumption into question, suggesting that recall can be a misleading measure of effectiveness when applied to positive emotive campaigns [20]. Nevertheless establishing how different campaigns are recalled is important in evaluating and comparing their impact.

In contrast with other media markets such as Australia, the UK provides an ideal context to evaluate the effects of different campaign types due to the wide variety of messaging and emotive content. This allowed us to explicitly compare population-level effects of exposure to both positive and negative emotive campaigns.

Using data from the International Tobacco Control (ITC) United Kingdom Survey, the present study sought to explore whether increased exposure to tobacco control campaigns results in increased probability of recall, and whether campaigns designed to elicit negative emotions achieve higher rates of recall than positive campaigns. In addition, we assessed duration of recall by testing the association between recall and campaign exposure in the 1–3 and 4–6 month periods before survey.

## Methods

### Survey methodology

Participants were drawn from waves 4 to 7 of the ITC United Kingdom Survey, a prospective longitudinal cohort study of adult smokers in the United Kingdom. Participants, who were aged ≥18 years and had smoked more than 100 cigarettes in their lifetime and provided informed consent, were interviewed annually by telephone between September and March of each survey year and asked a range of questions pertaining to smoking behaviour and attitudes [21]. After each survey, respondents received an incentive consisting of a £7 pharmacy voucher to encourage retention. New participants were recruited in each wave to replenish those lost to attrition or who had reported to have successfully discontinued smoking in two consecutive surveys. A more detailed description of the survey methodology is provided by Thompson et al. [22]. The study protocol was given ethical approval by the University of Waterloo and King’s College London.

### Sample characteristics

Of the 2,454 unique individuals residing in England who took part in at least one of waves four to seven of the ITC United Kingdom survey (from April 2005 to March 2009), our analysis included 1,968 participants (80.2%), who had provided outcome data at at least one of these waves and reported being a smoker in the previous wave of follow-up (which could include Waves 1–3). These individuals therefore had at least two waves of data, and between one and four observations (from Waves 4 to 7) were available for analysis on each individual. This provided 3,932 observations over four waves of follow-up, implying a mean of 2.0 observations per participant.

### Outcome measure

For the purposes of the analysis, participants who responded ‘*yes*’ to the question ‘*In the last 6 months, have you noticed advertising or information that talks about the dangers of smoking, or encourages quitting on television?*’ asked at each wave of the survey were considered to have recalled televised campaigns. This measure of spontaneous recall was operationalised as a binary variable.

### Campaign exposure

Exposure to government-funded televised tobacco control mass media campaigns, and to those run by charities including the British Heart Foundation and Cancer Research UK, was measured in GRPs, a standard measure of campaign reach giving a per capita measure of advertising exposure. For example, 1,000 GRPs could indicate that 100% of viewers were exposed to a given broadcast 10 times, or that 50% of viewers were exposed 20 times. On an individual level, actual exposure may vary according to a range of factors including television viewing frequency, channel and time of viewing. Per capita total monthly campaign exposure from April 2005 to March 2009 ranged from 0 to 1,051 GRPs, with a mean of 293.4. Total exposure over the period was 13,721 GRPs, including 809 GRPs for campaigns run by charities over the period studied. Although there was no discernible long-term upward or downward trend in GRPs, campaign exposure tended to peak in January of each year.

Campaigns were categorised according to a number of different features, including their emotive content, by two researchers using campaign creatives from the Central Office of Information and the UK Department of Health Tobacco Marketing Team, using a coding framework based on PRIME Theory [23]. There was complete concordance between the reviewers on theme, emotional content and delivery style, a third researcher resolved disagreement on the informational content of one advertisement. The framework and coding were validated by an eight-member focus group, a subset of the UK Centre for Tobacco and Alcohol Studies Smokers’ Panel. The methods employed are elaborated in further detail by Langley et al. [24].

**Figure 1:**
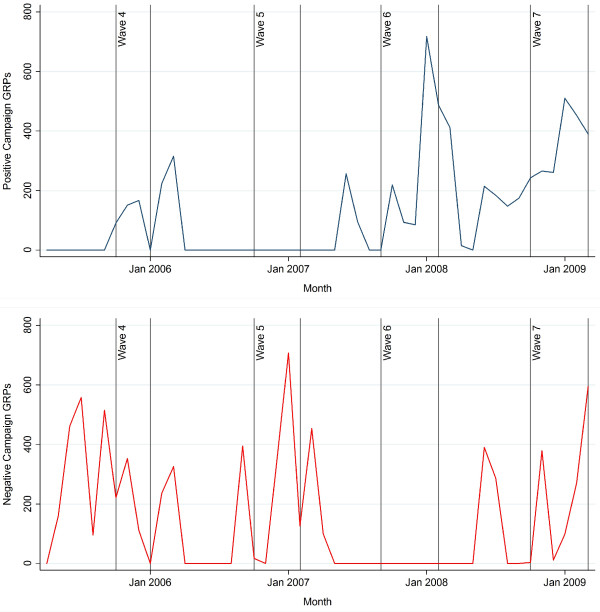
**Monthly GRPs, positive and negative emotive campaigns.** Legend: Monthly exposure to tobacco control campaigns measured in GRPs and data collection periods, positive (above) and negative (below) campaigns – Waves 4 to 9 (April 2005 to March 2009).

Campaigns were categorised as having either “positive” (eliciting happiness, satisfaction or hope) or “negative” (eliciting fear, sadness, guilt, anger or disgust) emotional content. Campaigns run by charities were added subsequent to the original coding and all of these campaigns, were considered to focus on the negative health effects of smoking and contained graphic imagery. Of all campaign GRPs in the study period, 42.4% were from campaigns featuring positive emotive content while 52.6% were from campaigns featuring negative emotive content. The remaining 5.0%, which contained neither positive nor negative emotive content and consisted of public information advertisements designed to raise awareness of smokefree legislation implemented in July 2007, were classified as “neutral”. As these campaigns did not depict the dangers of smoking or encourage cessation, and therefore did not relate to the outcome variable, they were removed from the analysis along with an additional 0.1 GRPs for which campaign creatives were unavailable. Monthly exposures to positive and negative campaigns, expressed in GRPs, are shown in Figure 1 for the entire study period — along with the data collection periods for each wave.

We generated variables measuring respondents’ exposure prior to each survey, expressed as the summed aggregations of GRPs over the 1–6 month period prior to the month of survey for each campaign type. To determine whether the duration of recall exceeded three months, we also generated measures of exposure to each campaign type in the 1–3 month and 4–6 month periods before participants were surveyed. While there was a negative correlation between positive and negative campaign exposures in the 1–6 months before survey (r = -0.517, p < 0.001), exposure to all campaign types in the 1–3 and 4–6 month periods before the date of survey were uncorrelated (r = 0.035, p = 0.822). This reflected the fact that campaigns generally occurred one at a time with only a small degree of overlap with other campaigns.

### Statistical analysis

Generalised estimating equations (GEE) for binary outcomes were used to estimate the effect of average exposure to different types of tobacco control advertising on the odds of campaign recall, allowing for an exchangeable correlation structure to account for the correlation of responses within individual participants. We used the *quasi-information criterion* (QIC) to determine the most appropriate correlation structure. This method allowed for multiple observations clustered within individuals to be analysed longitudinally, generating population-averaged effects [25]. While changing the working correlation structure had a limited impact on model outputs, model testing indicated that an exchangeable correlation structure was most appropriate. Cluster robust standard errors were used to calculate the variance. All analyses were carried out using STATA version 11.2.

All models adjusted for age, region of residence, level of education at recruitment, wave of recruitment (operationalised as categorical variables) and gender (as a binary variable) — all of which were all included *a priori* as potential confounders. An income variable was not included due to the relatively high number of missing responses. We additionally adjusted for potential seasonal effects by fitting an indicator variable for quarter of the year. However, this did not improve model fit or change effect estimates and was therefore not included in the final model.

Using data from the period April 2005 to March 2009, we regressed recall on exposure to all campaigns (Model 1) and on mutually adjusted exposure to campaigns with positive and negative emotive content in the 1–6 month period before the date of interview (Model 2). Campaign exposures were modelled as linear predictors. Duration of campaign recall was explored by testing the association between recall and campaign exposure by carrying out the same procedure for advertising exposure in both the 1–3 and 4–6 month periods before survey (Models 3 and 4), with a positive association between campaign recall and 4–6 month exposure for a given campaign type implying a duration of recall of over three months.

Finally, we tested the linearity of the association between GRPs and campaign recall by including both linear and quadratic terms, and then linear and square root terms, for GRPs in each model.

## Results

Table 1 shows the characteristics of the sample population, in addition to rates of recall by survey wave. Participants reported that they had recalled televised tobacco control campaigns in the previous six months in 3,269 out of 3,932 responses (83.1%).

**Table 1:** Final sample characteristics by wave of follow-up

**Variable**	**Wave**				**All waves**
**Category**	**4**	**5**	**6**	**7**	
	**(2005–2006)**	**(2006–2007)**	**(2007–2008)**	**(2008–2009)**	
Total	1077 (100)	960 (100)	981 (100)	914 (100)	3932 (100)
**Campaign recall**					
Yes	957 (88.9)	788 (82.0)	791 (80.6)	733 (80.2)	3269 (83.1)
No	120 (11.1)	172 (17.9)	190 (19.4)	181 (19.8)	663 (16.9)
**Age**					
18–24	46 (4.3)	40 (4.2)	48 (4.9)	43 (4.7)	177 (4.5)
25–39	290 (26.9)	262 (27.3)	258 (26.3)	220 (24.1)	1030 (26.2)
40–54	437 (40.6)	385 (40.1)	385 (39.2)	369 (40.4)	1576 (40.1)
55 +	304 (28.2)	273 (28.4)	290 (29.6)	282 (30.9)	1149 (29.2)
**Gender**					
Female	601 (55.8)	552 (57.5)	553 (56.4)	521 (57.0)	2227 (56.6)
Male	476 (44.2)	408 (42.5)	428 (43.6)	393 (43.0)	1705 (43.4)
**Level of Education**					
Low	376 (34.9)	325 (33.9)	287 (29.3)	252 (27.6)	1240 (31.5)
Middle	507 (47.1)	454 (47.3)	502 (51.2)	479 (52.4)	1942 (49.4)
High	194 (18.0)	181 (18.9)	192 (19.6)	183 (20.0)	750 (19.1)
**Region of residence**					
North East	63 (5.8)	49 (5.1)	56 (5.7)	49 (5.4)	217 (5.5)
Yorkshire	118 (11.0)	98 (10.2)	92 (9.4)	73 (8.0)	381 (9.7)
East Midlands	101 (9.4)	82 (8.5)	90 (9.2)	94 (10.3)	367 (9.3)
Eastern	104 (9.7)	102 (10.6)	102 (10.4)	96 (10.5)	404 (10.3)
London	153 (14.2)	147 (15.3)	165 (16.8)	142 (15.5)	607 (15.4)
South East	189 (17.5)	161 (16.8)	158 (16.1)	155 (17.0)	663 (16.9)
South West	95 (8.8)	102 (10.6)	107 (10.9)	99 (10.8)	403 (10.2)
West Midlands	109 (10.1)	95 (9.9)	96 (9.8)	103 (11.3)	403 (12.2)
North West	145 (13.5)	124 (12.9)	115 (11.7)	103 (11.3)	487 (12.4)
**Wave of recruitment**					
Wave 1 (2002–03)	653 (60.6)	437 (45.5)	316 (32.2)	221 (24.2)	1627 (41.4)
Wave 2 (2003–04)	86 (8.0)	52 (5.4)	38 (3.9)	28 (3.1)	204 (5.2)
Wave 3 (2004–05)	338 (31.4)	210 (20.9)	150 (15.3)	108 (11.8)	806 (20.5)
Wave 4 (2005–06)	N/A	261 (27.2)	168 (17.1)	108 (11.8)	537 (13.7)
Wave 5 (2006–07)	N/A	N/A	309 (31.5)	196 (21.4)	505 (12.8)
Wave 6 (2007–08)	N/A	N/A	N/A	253 (27.7)	253 (6.4)

### The effect of overall Six-month campaign exposure on probability of recall

Increased exposure to televised tobacco control campaigns was associated with higher odds of six-month campaign recall. As shown in Table 2, the odds of recall were increased by 26% (OR = 1.26, 95% CI: 1.12–1.41) for each additional 1,000 GRPs over the 1–6 month period before survey.

**Table 2:** Odds ratios for recall according to campaign type and period of exposure

**Model**	Campaign category GRPs*	**Period**	**OR**^**1 **^**(95% CI)**	**p**
			**(n = 3,932)**	
**1**	All Campaigns	1–6 months ago	1.26 (1.12–1.41)	< 0.001
**2**	Elicits Negative Emotions	1–6 months ago	1.41 (1.24–1.61)	< 0.001
	Elicits Positive Emotions	1–6 months ago	0.88 (0.71–1.09)	0.237
**3**	All Campaigns	1–3 months ago	1.51 (1.14–2.01)	0.004
	All Campaigns	4–6 months ago	1.08 (0.84–1.38)	0.566
**4**	Elicits Negative Emotions	1–3 months ago	1.63 (1.16–2.28)	0.005
	Elicits Negative Emotions	4–6 months ago	1.36 (1.04–1.77)	0.024
	Elicits Positive Emotions	1–3 months ago	0.71 (0.40–1.25)	0.237
	Elicits Positive Emotions	4–6 months ago	1.44 (0.56–3.71)	0.449

^1^adjusted for gender, age group, level of education, region of residence and cohort of recruitment, *in units of 1,000 GRPs.

### The effect of different types of campaign content on recall

While each additional 1,000 GRPs of per capita exposure to campaigns with negative emotive content resulted in a 41% increase in the odds of recall (OR = 1.41, 95% CI: 1.24–1.61), increased exposure to positive emotive campaigns did not result in increased recall (OR = 0.88, 95% CI: 0.71–1.09). These confidence intervals do not overlap, suggesting that increased exposure to negative campaigns has a greater effect on recall than positive campaigns.

### Duration of recall

While overall campaign exposure in the 1–3 month period before survey (OR = 1.51, 95% CI: 1.14–2.01) had a significant impact on recall, exposure in the 4–6 month period before survey did not (OR = 1.08, 95% CI: 0.84–1.38). Exposure to negative emotive campaigns in both the 1–3 month (OR =1.63, 95% CI: 1.16–2.28) and 4–6 month (OR = 1.36, 95% CI: 1.04–1.77) periods before each survey had a strong positive effect on recall while exposure to positive emotive campaigns in either period did not.

### Sensitivity analysis

There was no evidence to suggest that the relationship between exposure to any campaign type and recall, either in the 1–6, 1–3 or 4–6 months before survey, was non-linear. Refitting each model with additional quadratic and square root terms for exposure to each campaign type did not improve model fit in any instance.

## Discussion

We found that increased overall exposure to tobacco control campaigns resulted in increased odds of recall in the following six months. Furthermore, increased exposure to negative campaigns had a greater effect on recall than positive campaigns. Increased per capita exposure to negative emotive campaigns was positively associated with recall in both the 1–3 and 4–6 months following the campaign, implying that this effect on recall lasted more than three months after exposure. In contrast, higher exposure to positive campaign content was not associated with increased recall. These findings suggest that campaigns designed to elicit negative emotions towards smoking generate higher rates of recall.

One limitation of the present study is that GRP exposure data is averaged across the population and does not reflect individual levels of exposure as determined by time, duration and channel of viewing. A further potential limitation is that our coding of campaign content was conducted by only two researchers, and may therefore be subject to misclassification. However, the majority of elements in our coding framework were objective and validated using a focus group of smokers whose interpretations were highly comparable to ours [24].

In contrast to Dunlop et al. [14], we were unable to control for the timing of the launch phase of individual campaigns, which has been associated with higher rates of recall due to the increased salience of novel content. Furthermore, the nature of the question posed prevented us from evaluating prompted and unprompted recall of specific advertisements. Recall is not a rare outcome and therefore odds ratios will overestimate estimates of relative risk. Our analysis nevertheless enables a comparison of size of effect between campaign types.

In this study, we have analysed the impacts of campaign content on recall, rather than on measures of smoking cessation activity. The ITC survey collects a number of other potentially relevant outcomes, such as quit attempts, which have previously been used for analysis of the impact of mass media campaigns in Australia [17]. In the UK, ITC surveys during the period studied were approximately evenly-spaced and predominantly conducted in October and November; with 82.2% of responses occurring in these months. For behavioural outcome measures such as recent quit attempts in the last three months, for example, which have been shown to be strongly influenced by seasonal effects and to peak in January [26], there was a lack of data to adequately control for seasonal factors.

The strength of our study lies in the variability of tobacco control campaigns in the UK, allowing us to compare the effects of positive and negative emotive advertising content. While other studies have made comparisons between campaigns on delivery style and the presence of graphic content, ours is the first to directly evaluate the effects of different types of emotional content on campaign recall in adult smokers.

Classic marketing theory assumes that high levels of recall improve advertising effectiveness and that campaign recall provides a proxy measure of effective campaign exposure [18,19]. Heath and Hyder [20] have shown that recall can underestimate the effectiveness of positive emotive brand campaigns. High recall of negative emotive campaigns, seen in this and other studies [10,14,15] may also be a misleading indicator of their effectiveness.

Therefore, whilst our results support the hypothesis that negative campaign content is more effective in increasing recall, this does not necessarily imply that this translates into increased smoking cessation, behaviour change or improvements in other outcome measures. The length of time that campaigns were recalled contrasts with our previous findings which suggest that impacts on quitting behaviour may be limited to the immediate aftermath of the campaigns [4]. It is possible that campaigns will be recalled for a longer time after airing, but that campaign effects on quitting behaviours will be tied more closely to recent campaign exposure.

## Conclusion

In conclusion, our findings show that while increased exposure to negative tobacco control campaigns increased levels of self-reported recall, those with positive emotive content did not. Furthermore, this remained the case even when exposure had taken place more than three months previously.

However, further studies are needed to explore the role of campaign recall in modifying smoking cessation behaviours and determine whether negative emotive campaigns have greater impact on smoking prevalence and behavioural outcomes than other campaign types.

## Abbreviations

GEE: Generalised estimating equation; GRP: Gross rating point; ITC: International tobacco control policy evaluation project; QIC: *Quasi-information Criterion.*

## Competing interests

The authors declare that they have no competing interests.

## Authors’ contributions

SR completed the statistical analysis and was responsible for composing the manuscript. TL and SR prepared and cleaned the data. Campaigns were categorised by MS, TL and LS. SL, TL, MS, AG, LS, RH and AM contributed to further drafts and reviewed the text for important intellectual content. SL, MS, AG, AM, TL conceived the idea for the study. AG was involved in mass media data collection while GF and AM were among those responsible for the design and development of the ITC-4 study. SR, SL, TL, MS, AG, AM, LS, RH and GF gave final approval of the version of the manuscript to be published. SR is the guarantor for the study; SR, AM, SL, TS, MS, AG, LS, RH and GF had full access to all of the data in the study and take responsibility for the integrity of the data and the accuracy of the data analysis.

## Pre-publication history

The pre-publication history for this paper can be accessed here:

http://www.biomedcentral.com/1471-2458/14/432/prepub
